# Anthocyanin improves kidney function in diabetic kidney disease by regulating amino acid metabolism

**DOI:** 10.1186/s12967-022-03717-9

**Published:** 2022-11-05

**Authors:** Yi-Xi Li, Yong-Ping Lu, Donge Tang, Bo Hu, Ze-Yu Zhang, Hong-Wei Wu, Li-Jing Fan, Kai-Wen Cai, Chun Tang, Yi-Qing Zhang, Ling Hong, Jing-jing Dong, Bao-zhang Guan, Liang-Hong Yin, Yong Dai, Wei-bin Bai, Zhi-Hua Zheng, Ting Zhu

**Affiliations:** 1 Department of Nephrology, Center of Kidney and Urology, The Seventh Affiliated Hospital, Sun Yat-Sen University, Shenzhen, 518020 China; 2 Department of Nephrology, The First Affiliated Hospital of Jinan University, Guangzhou, 510632 China; 3 Guangdong Provincial Engineering Research Center of Autoimmune Disease Precision Medicine, The Second Clinical Medical College of Jinan University, The First Affiliated Hospital of Southern University of Science and Technology, Shenzhen People’s Hospital, Shenzhen, 518020 China; 4 Department of Nephrology, Guangzhou First People’s Hospital, Guangzhou, 510180 China; 5 Department of Food Science and Engineering, Institute of Food Safety and Nutrition, Guangdong Engineering Technology Center of Food Safety Molecular Rapid Detection, Jinan University, Guangzhou, 510632 China

**Keywords:** Diabetic nephropathy, Amino acid metabolism, Proteomics, Metabolomics, Chronic kidney disease

## Abstract

**Background:**

Diabetic kidney disease (DKD) is among the most important causes for chronic kidney disease. Anthocyanins (ANT) are polyphenolic compounds present in various food and play an important role in ameliorating hyperglycemia and insulin sensitivity. However, the effects of ANT in DKD are still poorly understood. This study aimed to investigate the effect of ANT (cyanidin-3-O-glucoside [C3G]) on the renal function of DKD, and whether the anti-DKD effect of ANT is related to metabolic pathways.

**Methods:**

To explore the role of ANT in DKD, we performed the examination of blood glucose, renal function, and histopathology. As for the mechanism, we designed the label-free quantification proteomics and nontargeted metabolomics analysis for kidney and serum. Subsequently, we revealed the anti-DKD effect of ANT through the bioinformatic analysis.

**Results:**

We showed that the fasting blood glucose level (− 6.1 mmol/L, *P* = 0.037), perimeter of glomerular lesions (− 24.1 μm, *P* = 0.030), fibrosis score of glomerular (− 8.8%, *P* = 0.002), and kidney function (Cystatin C: − 701.4 pg/mL, *P* = 0.043; urine creatinine: − 701.4 mmol/L, *P* = 0.032) were significantly alleviated in DKD mice after ANT treatment compared to untreated in the 20th week. Further, proteins and metabolites in the kidneys of DKD mice were observed to be dramatically altered due to changes in amino acid metabolism with ANT treatment; mainly, taurine and hypotaurine metabolism pathway was upregulated (*P* = 0.0001, t value = 5.97). Furthermore, upregulated tryptophan metabolism (*P* < 0.0001, t value = 5.94) and tyrosine metabolism (*P* = 0.0037, t value = 2.91) pathways had effects on serum of DKD mice as responsed ANT regulating.

**Conclusions:**

Our results suggested that prevention of the progression of DKD by ANT could be related to the regulation of amino acid metabolism. The use of dietary ANT may be one of the dietary strategies to prevent and treat DKD.

**Supplementary Information:**

The online version contains supplementary material available at 10.1186/s12967-022-03717-9.

## Background

Diabetic kidney disease (DKD) develops in more than 40% of patients with diabetes mellitus (DM) and is a principal leading cause for chronic kidney disease (CKD) globally [1]. DKD overlaps with pathological features, characterized by arteriolar hyalinosis and nodular glomerulosclerosis [2]. There is a considerable amount of complex mechanisms describing the development of DKD, including the accumulation of advanced glycation end-products and reactive oxygen species to trigger changes in glomerular [3]. Besides the continuous and durative existing of hyperglycemia, prior studies that have noted the importance of multiple codependent risk factors for DKD, such as hypertension, obesity, dietary factors, insulin resistance, and dyslipidemia [4]. The renin–angiotensin–aldosterone system (RAAS) inhibitors have been used for the management of DKD for approximately 20 years, however, RAAS inhibitors are associated with worse outcomes and adverse events including mild hyperkalemia, reversible hypotension, and acute kidney injury in the context of hypovolemia [5]. Mineralocorticoid receptor antagonists (MRAs) has become available on the treatment for DKD patients with refractory hypertension; however, their use is principally limited in virtue of their hyperkalemic effects [6]. Sodium-glucose linked transporter 2 inhibitor (SGLT-2i), as the basic treatment in DKD management, has a modest effect on reducing glycated hemoglobin (HbA_1c_) and robust clinical effects in cardiorenal outcomes [3] with inevitable risks of hypovolemia and ketosis [7]. The risk of DKD progression and death remains high because of the limitation of current therapeutic options. Therefore, developing new treatment strategies to delay the progression of DKD is necessary.

Anthocyanins (ANT) are a species of widely distributed flavonoid compounds, which are abundant in flowers, fruits, seeds, and plant leaves [8]. Notably, ANT exhibits superior bioavailability and antioxidative, antiangiogenic, antibacterial, anti-inflammatory, and antiatherosclerotic properties [9]. ANT shows a direct antioxidant action to protect DNA, proteins, and lipids from damage [10]. On the other hand, it is indirectly that ANT activates specific detoxification enzymes, such as glutathione reductase, glutathione peroxidase, and glutathione S-transferase, to reduce oxidative stress [11]. To data, various studies have demonstrated the efficancy of ANT to ameliorate hyperglycemia and insulin sensitivity and to protect β cells, increase secretion of insulin in diabetic mice [12]. Also, ANT has been found to exist the renal protection actions in diabetic mice which reduces urinary microalbumin and ameliorates renal lipotoxicity [13]. Further, ANT appears to be related to preventing glomerular angiogenesis by perturbing the Angpt-Tie-2 ligand–receptor system [14]. The mechanisms of kidney protection action are primarily related to their antioxidant properties; however, possible additional roles of enzymatic inhibition and metabolomic pathways are poorly studied.

In this study, we performed integrative label-free quantification proteomic and nontargeted metabolomic analyses of kidney and serum samples obtained from DKD mice to evaluate the effect of ANT, which provided a further understanding of the protection mechanisms of ANT in diabetes.

## Methods

### High purity C3G preparation

Cyanidin-3-O-glucoside (C3G) is the most abundant anthocyanin in the plant world. Black soybean peel (Anhui, China) was prepared as raw materials for C3G. The crude extraction of anthocyanin was according to the methods as described in previous research [15]. Following, C3G was seperated and purified from the crude extraction by ultra performance liquid chromatography-tandem mass spectrometry. Ultimately, the target fragment was identified as C3G, and the purity was more than 94.3%.

### Animals and research design

**Fig. 1:**
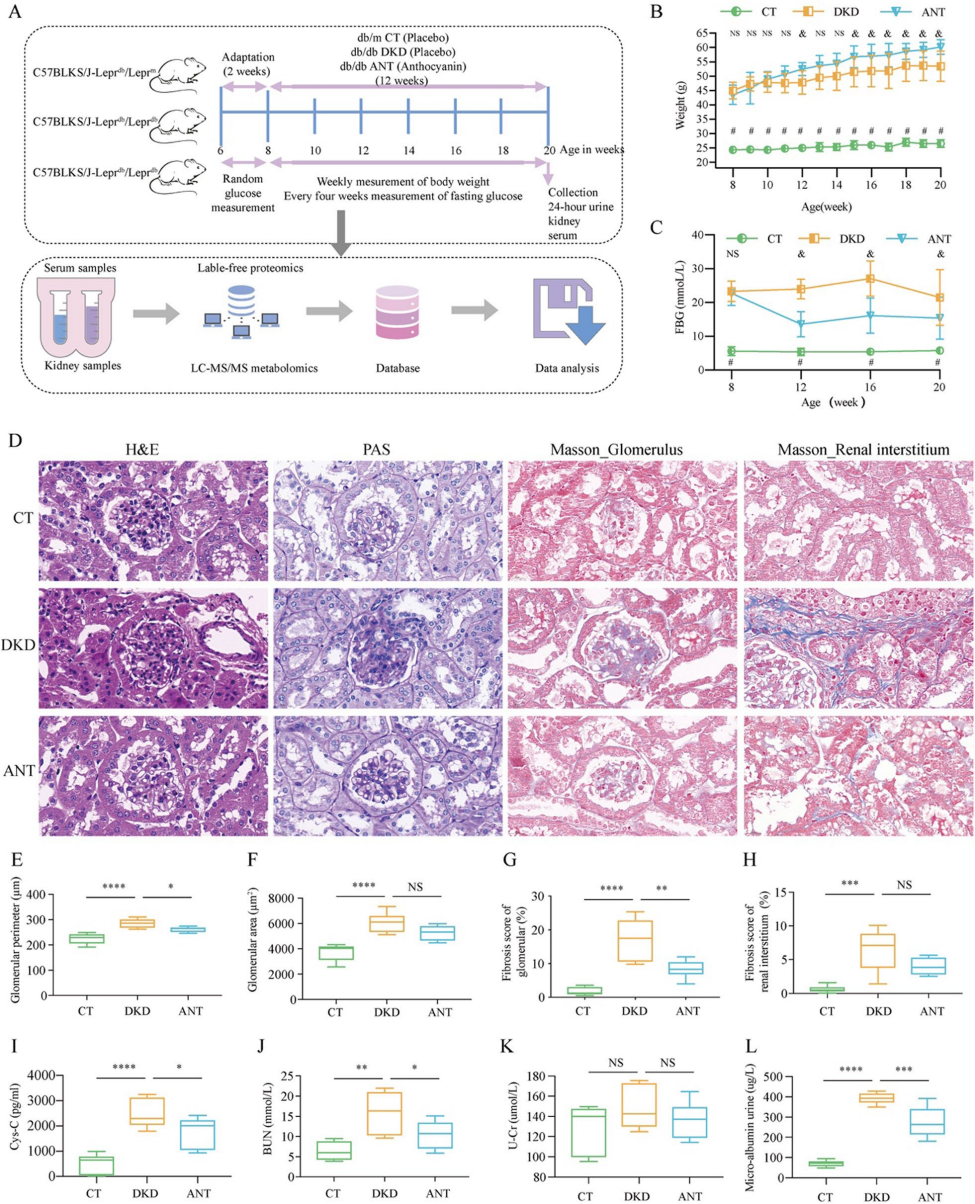
Study design and renal histopathological characteristics. The work flow of this study (**A.**) The weekly body weight of mice from 8- to 20-weeks (**B**) and the fasting blood glucose (FBG) at 8-, 12-, 16-, and 20-weeks (**C)** in the control group (CT), diabetic kidney disease group (DKD), and anthocyanin (ANT)-treated group all three groups. The green, orange, and blue nodes represent the CT, DKD, and ANT group, respectively (two-way ANOVA and multiple comparison using Tukey’s honest difference test, ^#^between the DKD vs. CT groups, *P* < 0.05; ^&^between the ANT vs. DKD groups, *P* < 0.05; *NS* not significant). (**D**) ANT improved renal dysfunction in diabetic mice. Representative photomicrographs of the kidney sections observed with hematoxylin&eosin (H&E), periodic acid–Schiff (PAS), and Masson staining (scale bar, 20 μm). Glomeruli in the kidney sections were visualized using H&E staining. The mesangial matrix was seen in the PAS staining. The interstitial fibers were visualized with Masson staining. The glomerular perimeter (**E**), area (**F**), and fibrosis score (**G)** and fibrosis score of renal interstitium (**H**) in three groups. The cystatin-C (**I**), blood urea nitrogen (**J**), urinary creatinine (**K**), and urinary microalbumin (**L**) in the three groups. The green, orange, and blue nodes represent the CT, DKD, and ANT groups, respectively (one-way ANOVA and multiple comparison using Holm-Sidak’s multiple comparisons test; ****P < 0.0001; ***P < 0.001; **P < 0.01; *P < 0.05; *NS* not significant)

Overall, 6-week-old male C57BLKS/J-Lepr^db^/Lepr^db^ mice (n = 22) and matched littermate C57BLKS/J-Lepr^db^/Lepr^m^ mice as normal control (CT group, n = 12) were purchased from Nanjing University Experimental Animal Center (Naijing, China). All mice were housed under a controlled SPF-grade environment (temperature: 22 ± 2℃; humidity: 55% ± 10%; with a 12 h/12 h light/dark cycle) with dietary drinking water available ad libitum. After two weeks of adaptive feeding with standard laboratory diet, the db/db mice were randomly divided into DKD group (model group) and ANT group (ANT-treated group) containing 11 mice each. The ANT group was administered with 10 mg/kg C3G per day by oral gavage for 12 weeks. In the CT and DKD groups, 10 mL/kg of sterile water was administered in the same way. From third week to fourteenth week, all mice were weighed once a week; whole blood was collected from tail veins after overnight fasting, and a portable glucometer (Accu-Chek, Switzerland) was used to measure the fasting blood glucose of all mice every four weeks. After 12 weeks treatment, 24-h urine samples were obtained from all mice using metabolic cages before the end of the experiment, and the urine samples were stored at − 80 °C. Further, the mice were anesthetized using intraperitoneal injection of 2,2,2-tribromoethanol (Thermo Fisher Scientific, MA, USA). The whole blood samples were obtained using cardiac puncture. Further, upper serum was collected and stored at − 80 °C. For the collected kidney specimens, half side of a kidney was snap-frozen in liquid nitrogen after weighting and stored at − 80 °C, whereas the other side of kidney was paraffin-embedded after fixing overnight in 4% paraformaldehyde. The research design was shown in Fig. 1A.

### Biochemical analyses and histopathological examination

The serum and urine samples were thawed at 4 °C before further analysis. Serum cystosin C levels were measured using a Mouse Cys-C (Cystatin C) ELISA Kit (E-EL-M3024, Elabscience, Wuhan, China). Urine creatinine (UCR) and microalbumin levels were measured using UCR enzyme-linked immunoassay (ELISA) kit (MM-44289M1, Elabscience, Wuhan, China) and MAU/ALB enzyme-linked immunoassay kit (MM-0705M1, Elabscience, Wuhan, China), respectively. Further, the urinary albumin to creatinine ratio (ACR) was calculated. The kidney samples were embedded in paraffin after 10% formalin fixed, and then cut into 5-µm sections. The sections were deparaffinized and rehydrated for further analysis. After, we performed the hematoxylin & eosin (H&E) staining, periodic acid–Schiff (PAS) staining, and Masson’s trichrome staining according to the manufacturer’s protocol. Following, images were captured using the EVOS XL Core Imaging System (Thermo Fisher Scientific, MA, USA).

### Proteomic analysis and data acquisition

For each kidney and serum samples, total protein was extracted, quantified, reduced, alkylated, digested to peptides, and desalted. Following, 2 µg peptides were carried out using the nano-UPLC coupled to the Q Exactive HFX Orbitrap instrument (Thermo Fisher Scientific, MA, USA) with the nano-electrospray ion source. Mobile phases were H_2_O with 0.1% formic acid (FA) and 2% acetonitrile (ACN) as phase A and 80% ACN with 0.1% FA as phase B. A reversed-phase column (Dr. Maisch, Germany) were used to seperated the peptides with a linear 120-min gradient (2–5% B for 2 min, 5–22% B for 88 min, 22–45% B for 26 min, 45–95% B for 2 min, and 95% B for 2 min). Data-dependent acquisition was performed with Orbitrap analyzer in positive modes at a resolution from 350 m/z to 1600 m/z for MS1. For MS2 spectra, the resolution was acquired with 15,000. The automatic gain control target was set to 50 ms and 3 × 10^6^ charges for MS1, and 110 ms and 1 × 10^5^ charges for MS2. The top 20 precursor ions were fragmented with higher energy collisional dissociation at an isolation window with 1.2 m/z and normalized collision energy (NCE) of 27. Only single-charged peaks with assigned charge states ≤6 were considered, and previously sequenced precursors were dynamically excluded for 45 s.

Raw MS files were performed with Proteome Discoverer software (Version 2.4.0.305) and searched against Mus musculus UniProt databases (UniProt-Mus musculus-10090-2020-10) using the built-in Sequest HT search engine. Carbamidomethyl was specified as a fixed modification, while acetylation and methionine oxidation at protein N-term were considered as variable modifications. We required a maximum of two missed cleavage sites, and less than 1% of false discovery rate for both peptide spectrum matches and protein group identifications. Peptide identification was performed with an initial precursor mass deviation of up to 10 ppm and a fragment mass deviation of 0.02 Da. Unique peptide and Razor peptide were used for protein quantification and total peptide amount for normalization. After that, 6355 proteins in kidney samples and 1673 proteins in serum samples were left and the remaining missing values were filled with half of minimum, shown in Additional file 2: Table S1 and Additional file 3: Table S2, respectively.

### Metabolomic analysis and data acquisition

After collection, each 50 mg kidney samples were weighted and added 1000 μL extract solution containing isotopically labelled internal standard mixture (methanol:acetonitrile:water in 2:2:1 ratio). Then, the kidney samples were homogenized at 35 Hz for 4 min and sonicated for 5 min in an ice-water bath with three repeated. At the same time, each 50 μL collected serum samples were added 200 μL extract solution containing isotopically labelled internal standard mixture (acetonitrile:methanol in 1:1 ratio), then, the serum samples were vortexed for 30 s and sonicated for 10 min at 4 °C. Following, all the kidney and serum samples were incubated for 1 h at − 40 °C and centrifuged at 14,000*g* for 15 min at 4 °C. All the resulting supernatant were collected to metabolomics profile analysis, respectively. 10 uL supernatant from each kidney and serum sample was pooled together to generate a quality control (QC) sample.

All the samples were analyzed by Vanquish (Thermo Fisher Scientific, MA, USA) coupled to Q Exactive HFX mass spectrometer (Thermo Fisher Scientific, MA, USA). All the metabolites were separated by an ACQUITY UPLC BEH Amide column (Waters, MA, USA) and analyzed in both positive and negative ion modes. The mobile phase A consisted of 25 mM/L ammonium acetate in water or 25 mM/L ammonium hydroxide in water for positive and negative modes, respectively. Mobile phase B was ACN. The elution gradient was set as follows: 0–0.5 min, 95% B; 0.5–7 min, 95–65% B; 7–8 min, 65–40% B; 8–9 min, 40% B; 9–9.1 min, 40–95% B; 9.1–12 min, 95% B. The flow rate was 0.5 mL/min and the injection volume was 3 μL. The Q Exactive HFX mass spectrometer was used to require MS/MS spectra on the information-dependent acquisition mode under the control of the acquisition software Xcalibur (v4.3, Thermo Fisher Scientific, MA, USA). The ESI source conditions were set as follows: sheath gas flow rate of 30 Arb; Aux gas flow rate of 25 Arb; capillary temperature for 350 °C; full MS resolution with 60,000; MS/MS resolution with 7500; collision energy of 10/30/60 in NCE mode; and spray voltage for 3.6 kV (positive) or − 3.2 kV (negative).

The obtained metabolomics raw data were converted to the mzXML format using ProteoWizard and processed with R package XCMS (v3.5, CA, USA). Prior to data analysis, peak pretreatments included identification, alignment, extraction, and integration. Then, the variability of the MS platform was monitored and adjusted based on the acquired QC spectra to make the data reproducible and reliable. The remaining peaks were annotated by comparison to retention time and mass to charge ratio (m/z) indexes in the library consisting of the online database of HMDB (www.hmdb.ca), KEGG (www.keg.jp) and an in-house-generated library (Biotree DB). Minfrac and cut-off are set as 0.5 and 0.3, respectively. In total, 9341 and 9761 peaks were detected in kidney samples under negative and positive ionisation modes, respectively. Also, 6938 and 8635 peaks were detected in serum samples under negative and positive ionisation modes, respectively. Metabolite peaks with relative SD of > 0.3 across QC samples and present in < 50% of samples were removed from the following analysis. After that, 5268 (negative mode) and 6676 (positive mode) metabolite features in kidney samples and 4276 (negative mode) and 5700 (positive mode) metabolite features in serum samples were left and the remaining missing values were filled with half of minimum. Following, a subset of 235 (negative mode) and 706 (positive mode) metabolites in kidney samples and 249 (negative mode) and 509 (positive mode) metabolites were annotated (MS2 score of > 0.8) using an in-house reference data, shown in Additional file 4: Table S3 and Additional file 5: Table S4, respectively. Finally, the positive-mode and negative-mode identified metabolites were concatenated for further analysis.

### Bioinformatic analysis

The normalized proteomic data were analyzed using TBtools software. Altered proteins with *P* < 0.05 and fold change (FC) > 1.5 or FC < 0.67 were considered differentially expressed proteins (DEPs). Metascape pathway enrichment analysis was used for the analysis of kidney and serum proteins; the minimum overlap was set as 3 and *P* value cutoff was set as 0.01.

The normalized metabolomic data were analyzed using metaboanalyt 5.0 software. Subsequently, the multivariate statistics were performed, in which the supervised partial least squares-based discriminant analysis (PLS-DA) was conducted to observe the metabolic profile of all subjects. This was done to find the abnormal outliers, assess the stability of QC samples, and characterize metabolic feature separation among the three groups. Orthogonal projections to latent structures discriminant analysis (OPLS-DA) was performed for characterizing metabolic feature separation among two groups. Q^2^ was the parameter for evaluating the predictability and interpretability of the OPLS-DA model. The variable importance in projection (VIP) was calculated based on PLS-DA to quantify the contribution of metabolites in the model and was used to screen differentially expressed metabolites (DEMs). The metabolites with VIP > 1, *P* < 0.05 (unpaired two-sided student’s t-tests), and FC > 1.5 or FC < 0.67 were selected as significant DEMs. The volcano plots and the hierarchically clustered heat map were plotted to visualize the feature of identified DEMs. The potentially altered metabolic pathways were assessed using the pathway analysis module on Metaboanalyst 5.0. The identified metabolites were classified according to the HMDB database and by counting the percentage of each metabolite category.

### Short time-series expression minor analysis

To identify the molecule signatures associated with ANT treatment, the short time-series expression minor (STEM) analysis was used to cluster protein and metabolite expression profiles from the CT, DKD, and ANT groups. The expression data were normalized using STEM clustering method. Minimum absolute expression change was set at 0.5 for molecule filtering, and the maximum correlation between any two model profiles was set as 0.9.

### Gene set variation analysis

All metabolite sets were downloaded from The Small Molecule Pathway Database (SMPDB). Gene set variation analysis (GSVA) was used to analyze Kyoto Encyclopedia of Genes and Genomes (KEGG) pathways of DEMs by R package GSVA and GSVA data. The selection criteria for significantly enriched KEGG pathway and differentially activated KEGG pathways were based on *P* < 0.05 and |t value|> 2, respectively.

### Immunofluorescence

The paraffin sections of kidney samples were permeabilized with 0.5% Trition at room temperature for 30 min. The supernatant was discarded, and the kidney sections were subjected to blocking with 5% BSA for 1 h. The kidney sections incubated with corresponding primary antibody, including anti-GGT1 (Affinity, # DF6610) (1: 200 dilution) and FMO5 (Proteintech, # 168641-AP) (1: 50 dilution), at 4 °C overnight. Then the kidney sections were incubated with the secondary antibody (1: 300 dilution) in dark at 4 °C for 2 h. After counterstained with DAPI, the kidney sections were observed under fluorescence microscope.

### Statistical analyses

All statistical analyses were performed using Prism (Graphpad, v.8.2.1). Pearson’s correlation test was used to describe the relationships between serum proteins or metabolites and kidney proteins or metabolites. Statistical significance was assessed using two-way ANOVA and multiple comparison using Tukey’s honest difference test, one-way ANOVA and multiple comparison using Holm-Sidak's multiple comparisons test, unpaired two-tailed student’s t-tests, moderated t-test, or permutation test where appropriate. Normally distributed data were expressed as mean ± standard deviation. *P* < 0.05 was considered statistically significant.

## Results

### Effects of ANT on biochemical parameters and renal histopathology in db/db mice

The body weight of db/db mice in the DKD group was much higher than that of db/m mice in the CT group from 8th week to 20th week (*P* < 0.05 for all; Fig. 1B). Further, the body weight of db/db mice in the ANT group was much higher than that of the DKD group after 15th week (*P* < 0.05; Fig. 1B), which kept the consistence with the previous studies that Jankowski, A. reported that anthocyanins from the red grapes showed inhibited loss of body mass [16], maqui berry increased body weight of diabetic rats [17], and anthocyanins and anthocyanidin suppressed the muscle weight loss by increasing oxidative stress-related cells [18]. The fasting blood glucose level of db/db mice in the DKD group was significantly increased as compared with the almost stable blood glucose level of the db/m mice in the CT group during the experiment after 8th week (*P* < 0.05 for all; Fig. 1C). ANT could ameliorate the elevated fasting blood glucose levels in the DKD group after 12th week (*P* < 0.05 for all; Fig. 1C). Therefore, ANT could reduce the fasting blood glucose level in DKD.

The effect of ANT on renal histopathology in DKD was studied. H&E staining of kidney sections revealed glomerular enlargement, edema of renal interstitium, and infiltrated inflammatory cells in db/db mice in the DKD group (Fig. 1D). The glomerular perimeter (Fig. 1E) and area (Fig. 1F) were increased in the DKD group compared with CT group (*P* < 0.0001). After ANT administration, the conditions of enlarged glomeruli and renal interstitium and infiltrated inflammatory cells were alleviated. The glomerular perimeter (Fig. 1E) was decreased in the ANT group compared with the DKD group (*P* < 0.05). PAS staining revealed increased glomerular cells and mesangial expansion in the DKD group. The glomerular lesions were significantly alleviated after ANT treatment (Fig. 1D). Masson-stained kidney tissues exhibited fibrosis in glomerulus and renal interstitium in the DKD group; however, the fibrosis was ameliorated under ANT treatment (Fig. 1D). The fibrosis scores of glomerulus (Fig. 1G) and renal interstitium (Fig. 1H) were increased in the DKD group compared with CT group (*P* < 0.0001). ANT could improve the elevated fibrosis score of glomerulus in the DKD group (*P* < 0.01; Fig. 1G). Therefore, ANT could alleviate glomerular lesions and fibrosis in DKD mice.

Moreover, cystatin-C (*P* < 0.0001; Fig. 1I), blood urea nitrogen (*P* < 0.01; Fig. 1J), and urinary microalbumin (Fig. 1L, P < 0.0001) were significantly elevated in the DKD group compared with the CT group. ANT could significantly reduce the elevated levels of cystatin-C (*P* < 0.05; Fig. 1I), blood urea nitrogen (*P* < 0.05; Fig. 1J), and urinary microalbumin (*P* < 0.001; Fig. 1L) in db/db mice. No significant difference was obtained in urinary creatinine level between the DKD vs. CT groups and ANT vs. DKD groups (*P* > 0.05; Fig. 1K). Therefore, ANT could alleviate renal function in db/db mice.

### Effects of ANT on the protein expression profile of db/db mice

**Fig. 2:**
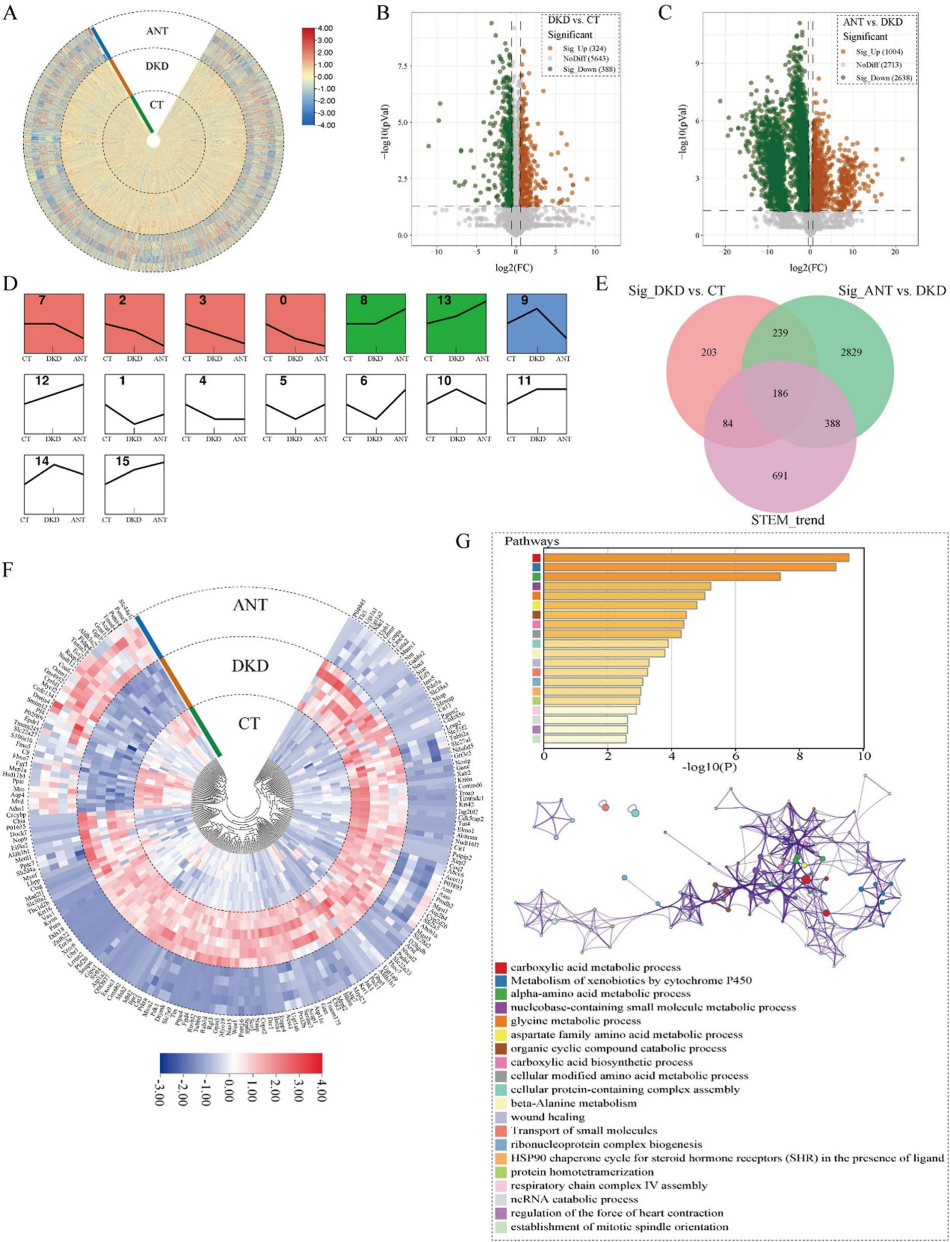
Effect of ANT on the protein expression profile in the kidney of db/db mice. (**A**) The heatmap of the expression of proteins in the kidney in the CT, DKD, and ANT groups. The expression of each protein was normalized. Volcano plots showing the differentially expressed proteins between the DKD vs. CT (**B**) and ANT vs. DKD (**C**) groups. (**D**) Renal proteomic profiles after STEM analysis. STEM analysis was applied to obtain the protein expression profiles across the CT, DKD, and ANT groups. Profile ID is shown at the top left corner of the profile. Lines in each profile represent the expression pattern of proteins across the three groups (permutation test, *P* < 0.05). (**E**) Venn diagram summarizing the differential and overlapping proteins among the DKD vs. CT groups, ANT vs. DKD groups, and STEM_trend (FC > 1.5 or FC < 0.67, unpaired two-sided student’s t-tests, *P* < 0.05). (**F**) Heatmap of the expression of ANT kidney trend proteins. (**G**) The function analysis of ANT kidney trend proteins in Metascape platform (*P* < 0.01)

Based on the label-free quantitative proteomics method, we detected 6355 proteins in the kidney in 6 db/db (CT), 6 DKD, and 5 ANT treated DKD (ANT) kidney samples (Fig. 2A). Among them, 712 and 3642 DEPs were identified between the DKD vs. CT and ANT vs. DKD groups, respectively (FC > 1.5 or FC < 0.67; unpaired two-sided student’s t-test, *P* < 0.05; Fig. 2B, C). To identify target proteins in the kidney regulated by ANT, we first applied a STEM analysis for the CT, DKD, and ANT groups and identified proteins in profiles 1, 5, 6, 9, 10, and 14 as trend proteins (Fig. 2D). Further, 186 kidney trend proteins in the ANT group were significantly differentially expressed in the kidneys between the DKD vs. CT and ANT vs. DKD groups and could be counteracted after ANT treatment (Fig. 2E, F). Among these proteins, increased levels of mannose-binding protein C (Mbl2), cystatin C (Cst3), and proto-oncogene vav (Vav1) might increase the risk of diabetic nephropathy. The kidney trend proteins in the ANT group were principally enriched in metabolic processes on the Metascape platform, including metabolism of carboxylic acids, alpha-amino acids, nucleobase-containing small molecules, glycine, aspartate family amino acids, cellular modified amino acids, and beta-alanine (*P* < 0.01; Fig. 2G).

**Fig. 3:**
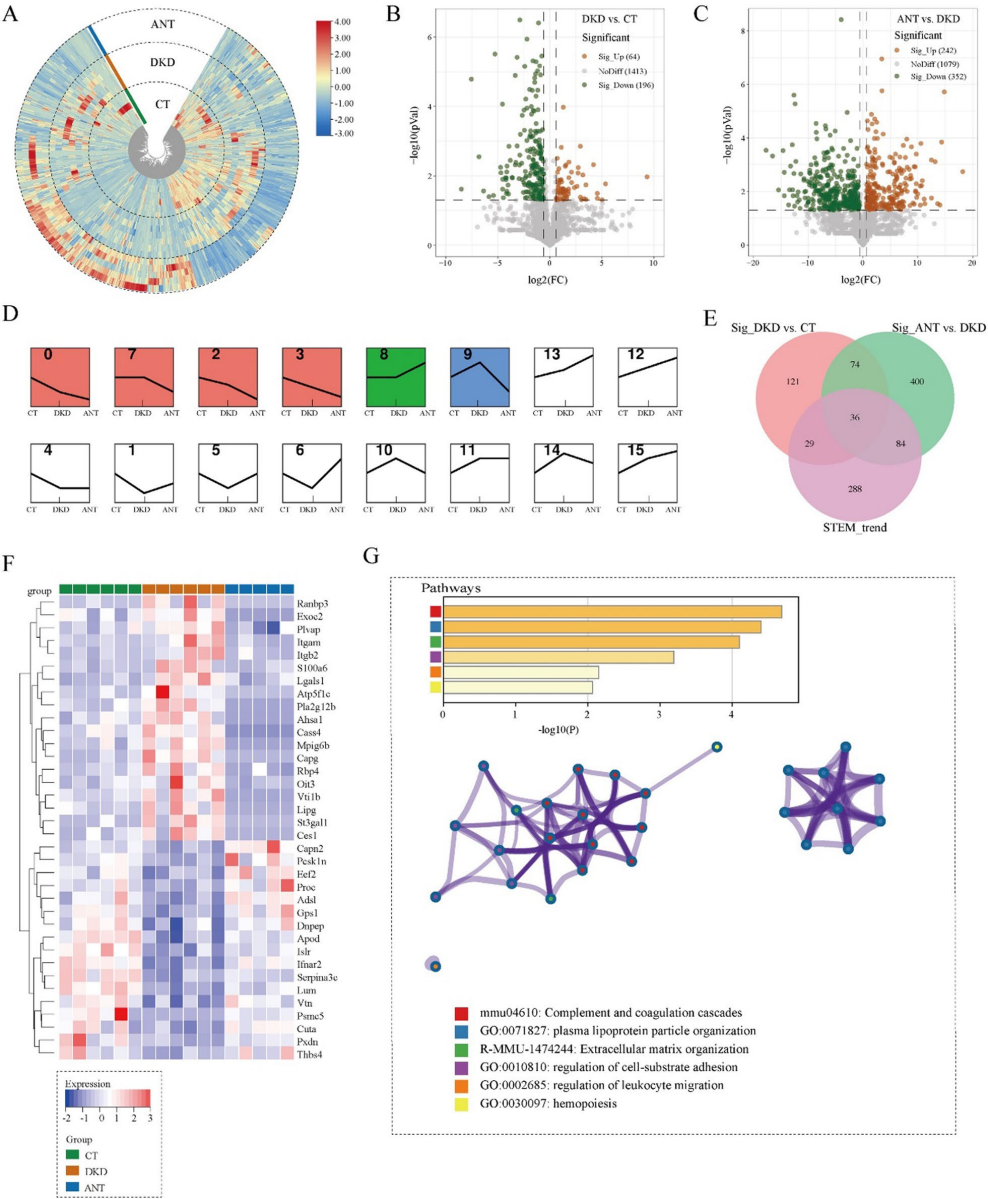
Effect of ANT on the protein expression profile in the serum of db/db mice. (**A**) The heatmap of the expression of serum proteins in the CT, DKD, and ANT groups. The expression of each protein was normalized. Volcano plots showing the differentially expressed proteins between the DKD vs. CT (**B**) and ANT vs. DKD (**C**) groups. (**D**) Serum proteomic profiles after STEM analysis. STEM analysis was applied to obtain the protein expression profiles across the CT, DKD, and ANT groups. Profile ID is shown at the top left corner of the profile. Lines in each profile represent the expression pattern of proteins across the three groups (permutation test, *P* < 0.05). (**E**) Venn diagram summarizing the differential and overlapping proteins among the DKD vs. CT groups, ANT vs. DKD groups, and STEM_trend (FC > 1.5 or FC < 0.67, unpaired two-sided student’s t-tests, *P* < 0.05). (**F**) The heatmap of the expression of ANT serum trend proteins in the three groups. (**G**) The function analysis of ANT serum trend proteins in Metascape platform (*P* < 0.01)

Meanwhile, we detected 1673 proteins in serum (Fig. 3A), among which 260 and 594 DEPs were identified between the DKD vs. CT and ANT vs. DKD groups, respectively (unpaired two-sided student’s t-test, *P* < 0.05, FC > 1.5 or FC < 0.67; Fig. 3B, C). We assessed target proteins in the kidney regulated by ANT. We observed 36 ANT serum trend proteins that were significantly differentially expressed in serum between the DKD vs. CT and ANT vs. DKD groups but could be counteracted after ANT treatment (Figs. 3D–F). Among these proteins, high levels of plasmalemma vesicle-associated protein (Plvap), integrin beta-2 (Itgb2), and retinol-binding protein 4 (Rbp4) might be correlated with increased severity of diabetic glomerulopathy. The ANT serum trend proteins were significantly enriched in complement and coagulation cascades, plasma lipoprotein particle organization, extracellular matrix organization, regulation of cell–substrate adhesion, regulation of leukocyte migration, and hemopoiesis on the Metascape platform (*P* < 0.01; Fig. 3G).

After comparing the DKD and CT groups, we observed 31 DEPs in both serum and kidney samples, including known diabetes-associated proteins such as Serpina3k, Lpl, C1qb, Lgals3, Ctsd, Pdgfra, Apoc3, Apoc1, Lifr, and Igfbp2, whereas 178 DEPs in both serum and kidney samples were detected between the ANT and DKD groups (Additional file 1: Fig. S1A). Only 5 identical DEPs (Capg, Pxdn, Mup20, Myh1, and C8g) were simultaneously detected for three groups in serum and kidney (Additional file 1: Fig. S1A). Additionally, correlation analysis revealed no significant correlation between circulating and renal tissue proteins in the CT, DKD, and ANT groups (Additional file 1: Fig. S2B–E), which suggested that the therapeutic effects of ANT on the renal and circulating protein profiles were mutually independent.

### Quality control of metabolomic analysis

Based on the nontargeted metabolomic analysis, we studied metabolic profile of kidney and serum in CT, DKD, and ANT three groups. In both positive and negative ion mode, the extracted ion chromatograms of the internal standard (IS) in quality control (QC) samples were adopted to assess the stability and reliability of the experimental method. The results revealed that the retention time and response intensity of IS in QC samples (kidney and serum) had good consistency (Additional file 1: Fig. S2D), indicating good stability of the data acquisition of mass spectrometers in this study. Meanwhile, Pearson correlation analysis was conducted on QC samples, and the correlations were high for all QC samples (the coefficient correlation was close to 1.00; Additional file 1: Fig. S2E, F), indicating the stability and high quality of the overall experimental method and data. In this study, we identified 886 (positive ion mode: 682 and negative ion mode: 204) and 724 (positive ion mode: 490 and negative ion mode: 234) metabolites from kidney and serum samples, respectively. The supervised PLS-DA model was used to assess the metabolic change trends for the QC, CTCT, DKD, and ANT four groups. 3D-PLS-DA score plots illustrated a significant separation trend between each pair of groups in kidney and serum samples (Additional file 1: Fig. S2G, H). It could be indicated that a substantial number of metabolites were differentially expressed in serum and kidney samples of the CT, DKD, and ANT three groups.

### The characteristics of the kidney and serum metabolomic landscapes in db/db mice

**Fig. 4:**
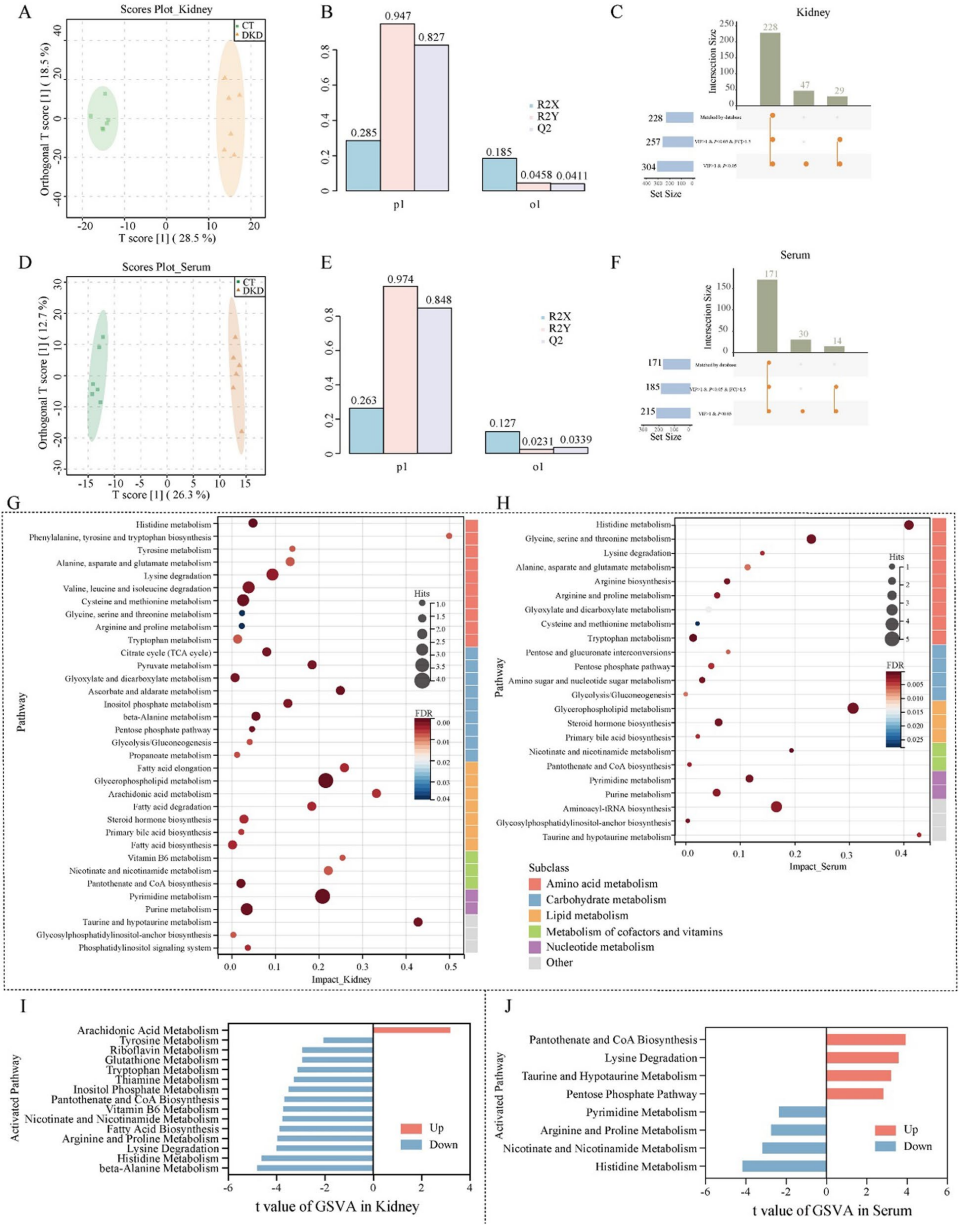
Metabolic profiles in the kidney and serum in DKD. The score plot of Orthogonal projections to latent structures discriminant analysis (OPLS-DA) for distinguishing metabolites in the kidney (**A**) and serum (**D**) between the DKD and CT groups. The OPLS-DA model for distinguishing metabolites in the kidney (**B**) and serum (**E**) between the DKD and CT groups. The upset plot for differentially expressed metabolites in the kidney (**C**) and serum (**F**) between the DKD and CT groups. Bubble plot for Kyoto Encyclopedia of Genes and Genomes (KEGG) pathway enrichment analysis in the kidney (**G**) and serum (**H**) (*P* < 0.05). The differentially activated KEGG pathways between the DKD and CT groups in the kidney (**I**) and serum (**J**). The red and blue bands represent the upregulated and downregulated pathways, respectively (moderated t-test, *P* < 0.05, |t value|> 2)

We compared kidney and serum metabolomics of the CT and DKD groups. In the kidney and serum, OPLS-DA model revealed noticeable differences in the distribution of metabolites between the CT and DKD groups (Fig. 4A and D) with Q^2^ = 0.827 and 0.848 (Fig. 4B and E),, respectively, which demonstrated that the OPLS-DA model was well predictive and reliable. According to the screening criteria of VIP > 1, *P* < 0.05, and FC > 1.5 or FC < 0.67, 257 and 185 significant DEMs were identified between the DKD and CT groups in the kidney and serum, respectively. Among them, 228 and 171 DEMs were finally matched by comparing the KEGG and HDMB databases in the kidney and serum, respectively (Fig. 4C and F). Additionally, correlation analysis revealed no significant correlation between circulating and renal tissue proteins in the CT and DKD groups (Additional file 1: Fig. S3C–E). To explore the aberrant metabolic pathway changes in DKD, we submitted 228 and 171 DEMs in kidney and serum, respectively, to Metaboanalyst 5.0 for KEGG pathway enrichment analysis; they were observed to be significantly enriched in 34 and 23 pathways in kidney and serum, respectively (FDR < 0.05; Fig. 4G and H), mainly involved in five types of metabolic abnormalities, including metabolism of amino acids, carbohydrates, lipids, cofactors and vitamins, and nucleotides. Further, we performed the GSVA analysis [19] for pathway activities between the two groups in the kidney and serum. In the kidney, arachidonic acid metabolism was ranked as the most sharply elevated pathway, and a decline in the activity of fatty acid biosynthesis (lipid metabolism pathway) was observed (moderated t-test, |t value|> 2; Fig. 4I). In addition, the alterations of many amino acid metabolism pathways activity were significantly decreased, including histidine metabolism, lysine degradation, arginine and proline metabolism, tryptophan metabolism, and tyrosine metabolism. Cofactor and vitamin metabolism pathways were significantly downregulated, including nicotinate and nicotinamide metabolism, vitamin B6 metabolism, and pantothenate and CoA biosynthesis. Carbohydrate metabolism pathways were significantly decreased, including inositol phosphate and beta-alanine metabolism. In serum (moderated t-test, |t value|> 2; Fig. 4J), lysine degradation pathway was elevated, and a decline in the activity of other amino acid metabolism pathways in the kidney and serum of the DKD group was observed. This highlighted the different alteration of metabolites in different biological entities for DKD pathogenesis.

### Effects of ANT on the metabolism expression profile of db/db mice

**Fig. 5:**
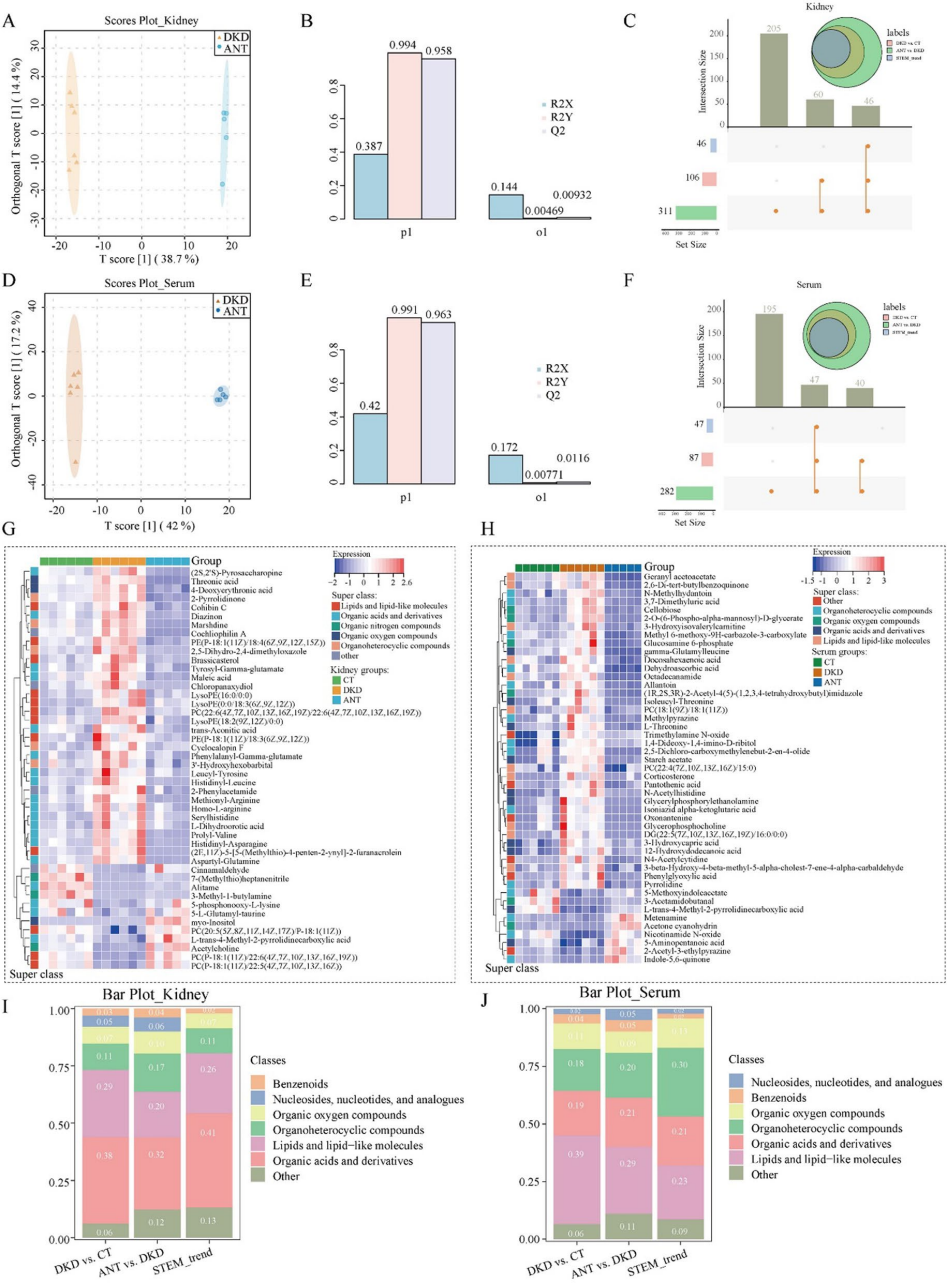
Effect of ANT on the metabolite expression profile in the kidney and serum of db/db mice. The score plot of OPLS-DA for distinguishing the metabolites in the kidney (**A**) and serum (**D**) between the ANT and DKD groups. The OPLS-DA model for distinguishing the metabolites in the kidney (**B**) and serum (**E**) between the ANT and DKD groups. The upset plot for differentially expressed metabolites among the DKD vs. CT groups, ANT vs. DKD groups, and STEM_trend in the kidney (**C**) and serum (**F**). The heatmap for the expression of ANT kidney trend metabolites (**G**) and ANT serum trend metabolites (**H**) in the three groups. Bar plot showing the percentage of differentially expressed metabolites among the DKD vs. CT groups, ANT vs. DKD groups, and STEM_trend in the kidney (**I**) and serum (**J**)

We constructed an OPLS-DA model in the kidney and serum to reveal noticeable differences in the distribution of metabolites between the DKD and ANT groups (Fig. 5A and D) with Q^2^ = 0.958 and 0.963 (Fig. 5B and E), respectively, which demonstrated that the OPLS-DA model was well predictive and reliable. Further, 335 and 305 significant DEMs were identified between the ANT vs. DKD groups in the kidney and serum according to the same screening criteria (VIP > 1, *P* < 0.05, and FC > 1.5 or FC < 0.67), respectively. Among them, 311 and 282 DEMs were finally matched by comparing the KEGG and HDMB databases in the kidney and serum, respectively (Additional file 1: Fig. S3A, B). Additionally, correlation analysis revealed no significant correlation between circulating and renal tissue proteins in the DKD and ANT groups (Additional file 1: Fig. S3G, H). To identify target kidney and serum metabolites regulated by ANT, we applied the STEM analysis for the CT, DKD, and ANT groups and identified metabolites in profiles 1, 5, 6, 9, 10, and 14 as STEM_trend metabolites (Additional file 1: Fig. S3F and I). The hierarchical cluster analysis was performed to 46 and 47 STEM_trend metabolites in the kidney and serum, respectively (Fig. 5G, H). Subsequently, we classified 228, 311, and 46 DEMs in the kidney between the DKD vs. CT, ANT vs. DKD, and STEM_trend metabolites according to the HMDB database (Fig. 5C) and observed that “organic acids and derivatives” category was enriched and “benzenoids” category was rare (Fig. 5I). Moreover, we classified 171, 282, and 47 DEMs in the serum between DKD vs. CT, ANT vs. DKD, and STEM_trend metabolites according to the HMDB database (Fig. 5F) and observed that “organoheterocyclic compounds” category was enriched and “nucleosides, nucleotides, and analogus” and “benzenoids” categories were rare in STEM_trend metabolites (Fig. 5J).

**Fig. 6:**
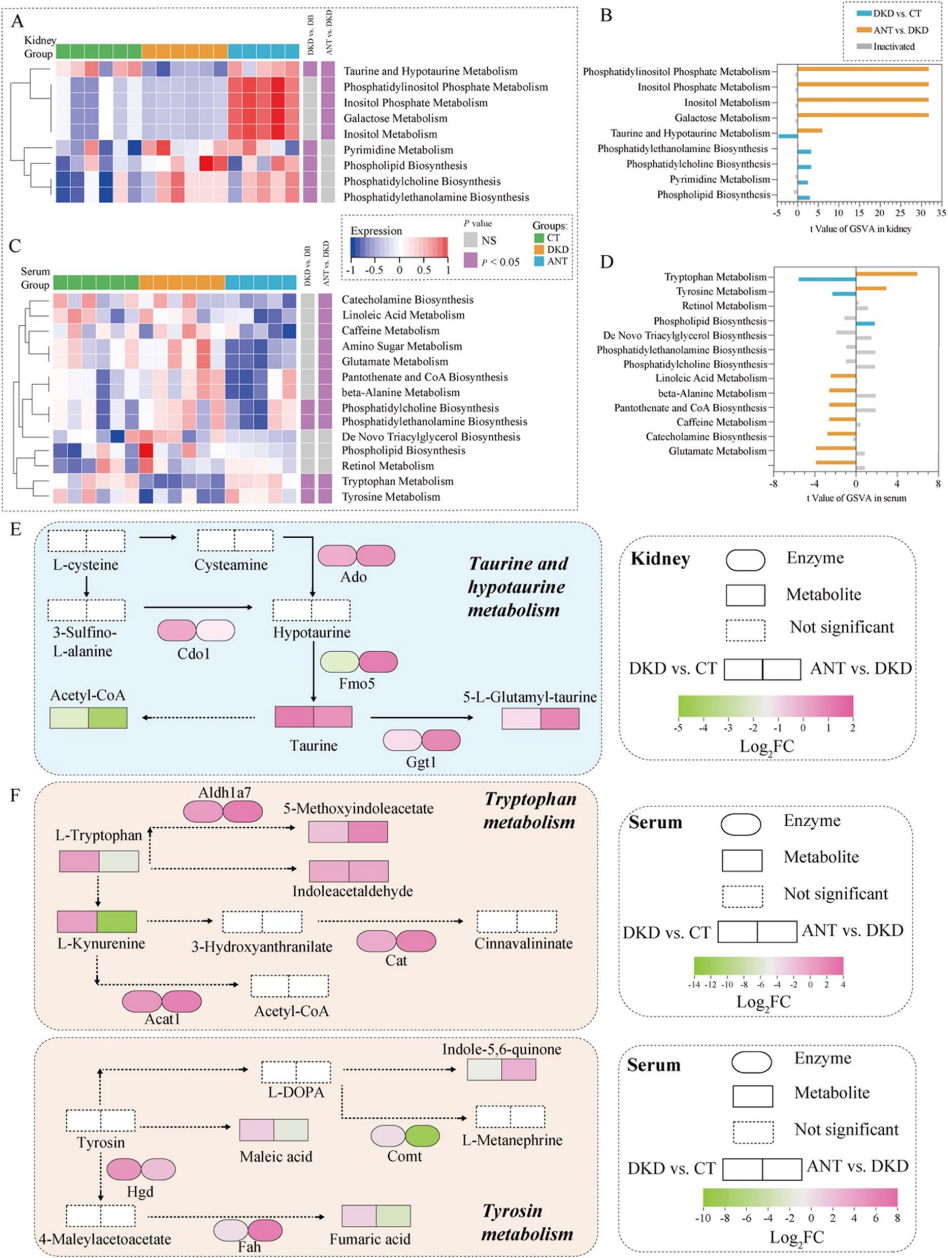
GSVA analysis of KEGG pathways for ANT trend metabolites and metabolic pathways altered by ANT in the kidney and serum. The enrichment score for nine KEGG pathways by GSVA analysis in the kidney (**A**) and serum (**C**) among the CT, DKD, and ANT three groups. The red and blue nodes represent enrichment score of KEGG pathways in each sample for three groups, respectively. The gray and purple nodes represent *P* value of KEGG pathways (moderated t-test, *P* < 0.05). The differentially activated KEGG pathways between the DKD vs. CT and ANT vs. DKD groups in the kidney (**B**) and serum (**D**). The blue and orange bands represent the activated KEGG pathways of the DKD vs. CT and ANT vs. DKD groups, respectively, and the gray bands represent the nonactivated KEGG pathways. (**E**) The diagram of a part of the taurine and hypotaurine metabolism pathway. The expression of taurine, 5-L-glutamyl-taurine, and acetyl-CoA is shown in (**E**). The enzymatic expression of Ado, Cdo1, Fmo5, and Ggt1 is shown in (**E**). Other metabolites, including hypotaurine, cysteamine, L-cysteine, and 3-sulfino-L-alanine, were not identified in the current study. (**F**) The diagram of a part of the tryptophan and tyrosine metabolism pathway. The expression of L-tryptophan, L-kynurenine, 5-methoxyindoleacetate, indoleacetaldehyde, maleic acid, fumaric acid, and indole-5,6-quinone is shown in (**F**). The expression of Aldh1a7, Acat1, Cat, Hgd, Comt, and Fah is shown in (**F**). Other metabolites, including 3-hydroxyanthranilate, cinnavalininate, Acetyl-CoA, tyrosine, L-DOPA, L-metanephrine, and 4-maleylacetoacetate, were not identified in the current study

We further performed GSVA analysis for 46 STEM_trend metabolites in the kidney and calculated the entire activity state for those enriched pathways. The taurine and hypotaurine metabolism pathway was significantly altered in both the DKD vs. CT and ANT vs. DKD groups **(**moderated t-test, *P* < 0.05; Fig. 6A**)**; the pathway was downregulated between the DKD and CT groups and upregulated between the ANT and DKD groups **(**|t value|> 2; Fig. 6B**)**. For taurine and hypotaurine metabolism, we observed that taurine and 5-L-glutamyl-taurine were upregulated and acetyl-CoA was downregulated in kidney after ANT treatment (Fig. 6E). And then we validated the expression of Fmo5 and Ggt1 in kidney by immunofluorescence. As a result, Fmo5 and Ggt1 immunostaining was present in normal renal tubular (CT group) in a perinuclear pattern and was localized mainly to tubular epithelial cells (Additional file 1: Fig. S4A and C), which is consistent with previous studies that Fmo5 are primarily localized to the distal tubules of the cortex, proximal tubules and the collecting ducts of the medulla [20], and Ggt1 is expressed at high levels in renal proximal tubules [21]. Then, we found that immunostaining for Fmo5 and Ggt1 confirmed the proteomics data (Fig. 2F) and showed that significantly lower Fmo5 (Additional file 1: Fig. S4B, *P* < 0.05, one-way ANOVA and multiple comparison using Holm-Sidak’s multiple comparisons test) and slightly lower Ggt1 levels (Additional file 1: Fig. S4D) were observed in DKD group compared to CT group. These data also indicated that following treatment with ANT there was a significantly increase in immunostaining for Fmo5 (Additional file 1: Fig. S4B, *P* < 0.05, one-way ANOVA and multiple comparison using Holm-Sidak’s multiple comparisons test) and relatively higher Ggt1 in kidney tissues of db/db mice (Additional file 1: Fig. S4D). The results of these two critical enzymes in taurine and hypotaurine metabolism pathway which confirmed the vital role of this pathway in ameliorating renal function under ANT treatment in DKD.

Moreover, we performed GSVA analysis for 47 STEM_trend metabolites in the serum. The phosphatidylcholine and phosphatidylethanolamine biosynthesis pathways were significantly altered between the DKD vs. CT and ANT vs. DKD groups **(**moderated t-test, *P* < 0.05; Fig. 6C**)**; the two pathways were upregulated between the DKD and CT groups and downregulated between the ANT and DKD groups **(**|t value|> 2; Fig. 6D**)**. Additionally, tryptophan and tyrosine metabolism pathways were significantly altered between the DKD vs. CT and ANT vs. DKD groups **(**moderated t-test, *P* < 0.05; Fig. 6C**)**; the two pathways were downregulated between the DKD and CT groups and upregulated between the ANT and DKD groups **(**|t value|> 2; Fig. 6D**)**. In tryptophan metabolism, we observed that L-tryptophan and L-kynurenine were downregulated, and 5-methoxyindoleacetate and indole-3-acetaldehyde were upregulated in serum after ANT treatment (Fig. 6F). In tyrosine metabolism, maleic acid and fumaric acid were downregulated, and indole-5,6-quinone was upregulated in serum after ANT treatment (Fig. 6F).

## Discussion

In this study, we performed label-free quantification proteomics and LC–MS/MS-based metabolomic analyses to explore the underlying mechanism of alleviation of kidney damage in DKD by ANT. Proteomic and metabolomic analyses revealed that ANT effectively altered the proteomic and metabolic phenotypes of the kidney and serum in db/db mice. We detected many DKD-associated serum and kidney proteins reported in previous studies, validating our approach. We observed that ANT kidney trend proteins and metabolites were enriched in amino acid metabolism, mainly in taurine and hypotaurine metabolism; ANT serum trend proteins were enriched in complement and coagulation cascades, and ANT serum trend metabolites were enriched in tryptophan and tyrosine metabolism.

A great deal of previous studies have considered ANT, the single compound C3G, has potential effects of antioxidation and anti-inflammation, which was put down to the capacity for chelate ions of bivalent metals [22] and inhibiting the proinflammatory cytokines [23]. In addition, researchers attempted to evaluate the impact of anthocyanins or C3G for preventing diabetes-associated kidney injury. The effect of anti-inflammation of C3G is in part induced to suppressing the liver X receptor alpha (LXRα)-stimulated nuclear translocation of nuclear factor-κB (NFκB), further, to reducing the levels of proinflammatory cytokines [24]. C3G from black rice can also showed the protective effects of DKD by regulating the transforming growth factor β1 (TGF-β1)/Smad pathway in a rat model of DKD [25]. Further, C3G alleviated the dysfunction of glucose and lipid metabolism and involved in the renal protection via the regulation of glutathione pool in db/db mice [26]. Consistent with the literature, our research found that anthocyanins C3G from black soybean peel could also decreased the blood glucose in DKD and ameliorate renal function. And we firstly found anthocyanins may regulate amino acid metabolism to participate in the kidney protection effects.

Taurine and hypotaurine have displayed varying degrees of antioxidative properties and are reported to lower diabetes-related hyperglycemia [27]. Further, taurine and related compounds protected red blood cells against diabetes-induced membrane damage, including reduced the formation of intracellular malondialdehyde and oxidized glutathione, and decreased the activities of reduced glutathione and antioxidative enzyme in diabetic erythrocytes [28]. Taurine also could be used as an additional intracellular osmolyte to maintain osmotic balance. A previous study [29] reported that hyperglycemia results in an increase in cerebral taurine uptake mediated by increased activity of the β-amino acid carrier system. Interestingly, the insulin was observed to prevent adaptive increase in brain taurine uptake in diabetic rats owing to maintaining euglycemia instead of the effect by insulin itself [29]. In our study, we observed that ANT could improve the acitivity of taurine and hypotaurine metabolism pathway in the kidney (Fig. 6A), which might play a role in preventing kidney damage from hyperglycemia.

As shown in Fig. 6C, ANT could upregulate the tryptophan metabolism pathway in serum in db/db mice. As one of the eight essential amino acids, the levels of tryptophan in urine [30] were reduced in patients with DKD. A prior study that have noted the relation of the decreased tryptophan to a rapid reduction in estimated glomerular filtration rate (eGFR) [31]. Moreover, what we known about the tryptophan effect of renal oxidation stress is largely based on variable hexamer structures and raised proteolytic degradation [32]. A number of studies have established that a majority of tryptophan (95%) indicated that can be metabolized through kynurenine pathway to produce various downstream metabolites [33]. What stands out for these downstream metabolites is exerting diverse even opposite roles in biological processes, such as apoptosis, inflammation, gluconeogenesis, and redox homeostasis [34]. In previous study, kynurenine and kynurenic acid showed be negatively correlated with eGFR [35]. Further, various studies have assessed the toxic properties of accumulated kynurenine derivatives that, binded with insulin to form excitotoxic complexes, may lead to systemic disorders, including insulin resistance, blood presure changed, and renal damage [36]. Previous studies demonstrated that the accumulated kynurenine metabolites may take part in the dysfunction of kidney cell bringing about kidney failure [34]. In the present study, we observed that ANT could decrease L-kynurenine levels in serum for db/db mice, suggesting that ANT may play a renoprotection role by regulating the kynurenate synthesis.

Due to tyrosine only synthesized by the hydroxylation of phenylalanine, it is recognized as a semiessential or conditionally indispensable amino acid [37]. Previous studies have explored the relationships between tyrosine and CKD that the level of tyrosine is dereceased in plasma [38]. The kidney is a major source of tyrosine. The kidney release of tyrosine was reduced in CKD patients, and directly correlated with its circulating levels, indicating that inhibited kidney production of tyrosine plays a vital role in depresssing circulating tyrosine [39]. What is surprising is that tyrosine metabolism turbulence is associated with insulin resistance [40]. In this study, ANT could upregulate tyrosine metabolism in serum in db/db mice. Further, nitrotyrosine (NT) is formed by using peroxynitrite to nitrificate tyrosine residues in proteins. It is somewhat interesting that NT was noted to participate in processes including inflammation, nitric oxide-induced oxidative stress, and cell damage [41]. A relationship exists between elevated levels of NT and the development of diabetes and its complications, reason of which is that NT could disturb renal function and worsen kidney pathology in diabetic rats [42]. Also, there are similarities between the finding for increased levels of NT in patients with DKD [43] and those described by Chew (2010). Moreover, a possible explanation for this might be that NT promotes the expression of inflammatory cytokines in glomerular mesangial cells to induce and aggravate kidney inflammatory damage [44]. It can therefore be assumed that ANT alleviated the nephropathy in part though activation of tyrosine metabolism pathway which inhibiting oxidative stress and reducing inflammatory cytokines.

## Conclusions

In conclusion, our study revealed multiple ameliorative effects of ANT in DKD including supressing fasting blood glucose, the improvement of renal function and histopathological changes in the kidney. The underlying mechanisms may be related to metabolic and proteomic reprogramming that upregulates metabolism of taurine, hypotaurine, tryptophan, and tyrosine. Dietary ANT may be a potential regulator of amino acid metabolism and can ameliorate renal function and morphology. Collectively, this study revealed potential clinical impact of use of dietary ANT in the prevention and treatment of DKD.

## Supplementary Information

**Additional file 1: Figure S1. **The correlation of differentially expressed proteins in the kidney and serum. (**A**) Venn plot showing the intersection of significantly altered proteins (FC > 1.5 or FC < 0.67 and *P* < 0.05) in the kidney and serum samples. Scatter plot showing the Pearson’s correlation of combined DEPs between serum and kidney proteins in the CT (**B**) and DKD ( **C**) groups. Scatter plot showing the Pearson’s correlation of combined DEPs between serum and kidney proteins in the DKD (**D**) and ANT (**E**) groups. **Figure S2. **The quality control (QC) of metabolomic profiling in the kidney and serum. The extracted ion chromatograms (EIC) of the internal standard (IS) in kidney QC samples in negative (**A**) and positive (**B**) ion model. The EIC of the IS in serum QC samples in negative (**C**) and positive (**D**) ion model. Pearson correlation analysis for QC samples in the kidney (**E**) and serum (**F**). 3D-PLS-DA score plots exhibited a significant separation trend among the CT, DKD, ANT, and QC groups in the kidney (**G**) and serum (**H**). **Figure S3. **Metabolic profile in the kidney and serum of the ANT group. The upset plot for differentially expressed metabolites in the kidney (**A**) and serum (**B**) between the ANT and DKD groups. (**C**) Venn plot showing the intersection of significantly altered metabolites (FC > 1.5 or FC < 0.67, and *P* < 0.05) in the kidney and serum samples. Scatter plot showing the Pearson’s correlation of combined DEMs between serum and kidney metabolites in the CT (**D**) and DKD (**E)** groups. Scatter plot showing the Pearson’s correlation of combined DEMs between serum and kidney metabolites in the DKD (**G**) and ANT (**H**) groups. The kidney (**F**) and serum (**I**) metabolites profiles after STEM analysis. STEM analysis was applied to obtain the metabolite expression profiles across the CT, DKD, and ANT groups. Profile ID is shown at the top left corner of the profile. Lines in each profile represent the expression pattern of proteins across the three groups (permutation test, *P* < 0.05). **Figure S4.** Immunostaining assays of ANT-treated kidney samples. Immunofluorescence revealed the expression of (**A**) Fmo5 and (**C**) Ggt1 levels in kidney tissues for CT, DKD, and ANT groups (blue fluorescence for DAPI; red immunofluorescence for Fmo5 or Ggt1; and scale bar as 20 µm). The immunostaining average positive area ratio of (**B**) Fmo5 and (D) Ggt1. The results of the statistical analysis are presented in bar charts (mean ± SD), by one-way ANOVA and multiple comparison using Holm-Sidak’s multiple comparisons test. **** *P* < 0.0001 compared with CT; ## *P *< 0.005 compared with DKD; ns, not significant.

**Additional file 2: Table S1.** The identified kidney proteins in CT, DKD, and ANT groups.

**Additional file 3: Table S2.** The identified serum proteins in CT, DKD, and ANT groups.

**Additional file 4: Table S3.** (A) The identified kidney metabolites under negative model in CT, DKD, and ANT groups. (B) The identified kidney metabolites under positive model in CT, DKD, and ANT groups.

**Additional file 5: Table S4.** (A) The identified serum metabolites under negative model in CT, DKD, and ANT groups. (B) The identified serum metabolites under positive model in CT, DKD, and ANT groups.

## Data Availability

The datasets supporting the conclusions of this article are available from the authors upon reasonable request.

## Abbreviations

DKD: Diabetic kidney disease
ANT: Anthocyanins
C3G: Cyanidin-3-O-glucoside
CKD: Chronic kidney disease
RAAS: Renin–angiotensin–aldosterone system
MRAs: Mineralocorticoid receptor antagonists
SGLT-2i: Sodium-glucose linked transporter 2 inhibitor
HbA_1c_: Glycated hemoglobin
Cys-C: Cystatin C
UCR: Urine creatinine
ELISA: Enzyme-linked immunoassay
FBG: Fasting blood glucose
ACR: Albumin to creatinine ratio
H&E: Hematoxylin & eosin
PAS: Periodic acid–Schiff
FA: Formic acid
ACN: Acetonitrile
NCE: Normalized collision energy
QC: Quality control
DEPs: Differentially expressed proteins
FC: Fold change
PLS-DA: Partial least squares-based discriminant analysis
OPLS-DA: Orthogonal projections to latent structures discriminant analysis
VIP: Variable importance in projection
DEMs: Differentially expressed metabolites
STEM: Short time-series expression minor
SMPDB: The Small Molecule Pathway Database
KEGG: Kyoto Encyclopedia of Genes and Genomes
GSVA: Gene set variation analysis
