# Supplementary material for: Anthocyanin improves kidney function in diabetic kidney disease by regulating amino acid metabolism
Source: J Transl Med. 2022 Nov 5;20:510. doi: 10.1186/s12967-022-03717-9 (PMC9636632; doi:10.1186/s12967-022-03717-9)
Supplement: Supplementary file 1 — Additional file 1: Figure S1. The correlation of differentially expressed proteins in the kidney and serum. (A) Venn plot showing the intersection of significantly altered proteins (FC > 1.5 or FC < 0.67 and P < 0.05) in the kidney and serum samples. Scatter plot showing the Pearson’s correlation of combined DEPs between serum and kidney proteins in the CT (B) and DKD ( C) groups. Scatter plot showing the Pearson’s correlation of combined DEPs between serum and kidney proteins in the DKD (D) and ANT (E) groups. Figure S2. The quality control (QC) of metabolomic profiling in the kidney and serum. The extracted ion chromatograms (EIC) of the internal standard (IS) in kidney QC samples in negative (A) and positive (B) ion model. The EIC of the IS in serum QC samples in negative (C) and positive (D) ion model. Pearson correlation analysis for QC samples in the kidney (E) and serum (F). 3D-PLS-DA score plots exhibited a significant separation trend among the CT, DKD, ANT, and QC groups in the kidney (G) and serum (H). Figure S3. Metabolic profile in the kidney and serum of the ANT group. The upset plot for differentially expressed metabolites in the kidney (A) and serum (B) between the ANT and DKD groups. (C) Venn plot showing the intersection of significantly altered metabolites (FC > 1.5 or FC < 0.67, and P < 0.05) in the kidney and serum samples. Scatter plot showing the Pearson’s correlation of combined DEMs between serum and kidney metabolites in the CT (D) and DKD (E) groups. Scatter plot showing the Pearson’s correlation of combined DEMs between serum and kidney metabolites in the DKD (G) and ANT (H) groups. The kidney (F) and serum (I) metabolites profiles after STEM analysis. STEM analysis was applied to obtain the metabolite expression profiles across the CT, DKD, and ANT groups. Profile ID is shown at the top left corner of the profile. Lines in each profile represent the expression pattern of proteins across the three groups (permutation test, P < 0.05). Figu [file 12967_2022_3717_MOESM1_ESM.docx]

**Additional file 1**


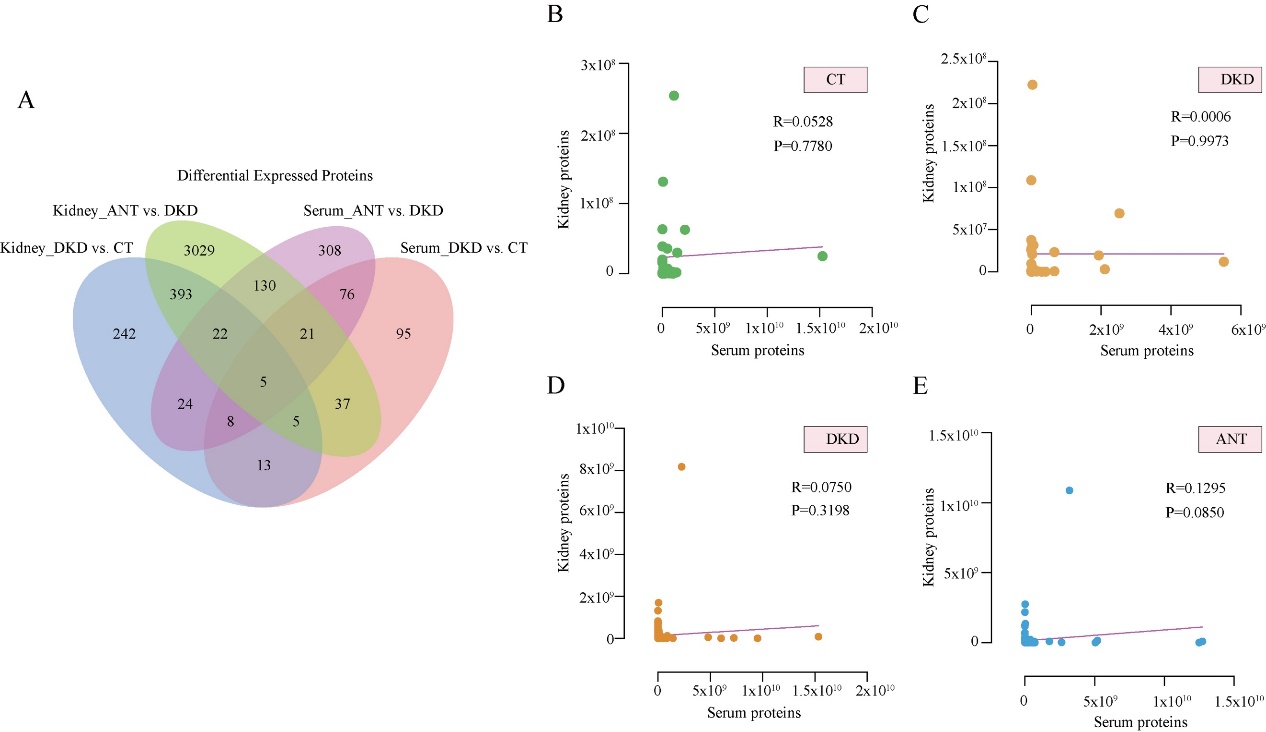


**Figure S1. The correlation of differentially expressed proteins in the kidney and serum**

(A) Venn plot showing the intersection of significantly altered proteins (FC > 1.5 or FC < 0.67 and *P* < 0.05) in the kidney and serum samples. Scatter plot showing the Pearson’s correlation of combined DEPs between serum and kidney proteins in the CT (B) and DKD (C) groups. Scatter plot showing the Pearson’s correlation of combined DEPs between serum and kidney proteins in the DKD (D) and ANT (E) groups.

**
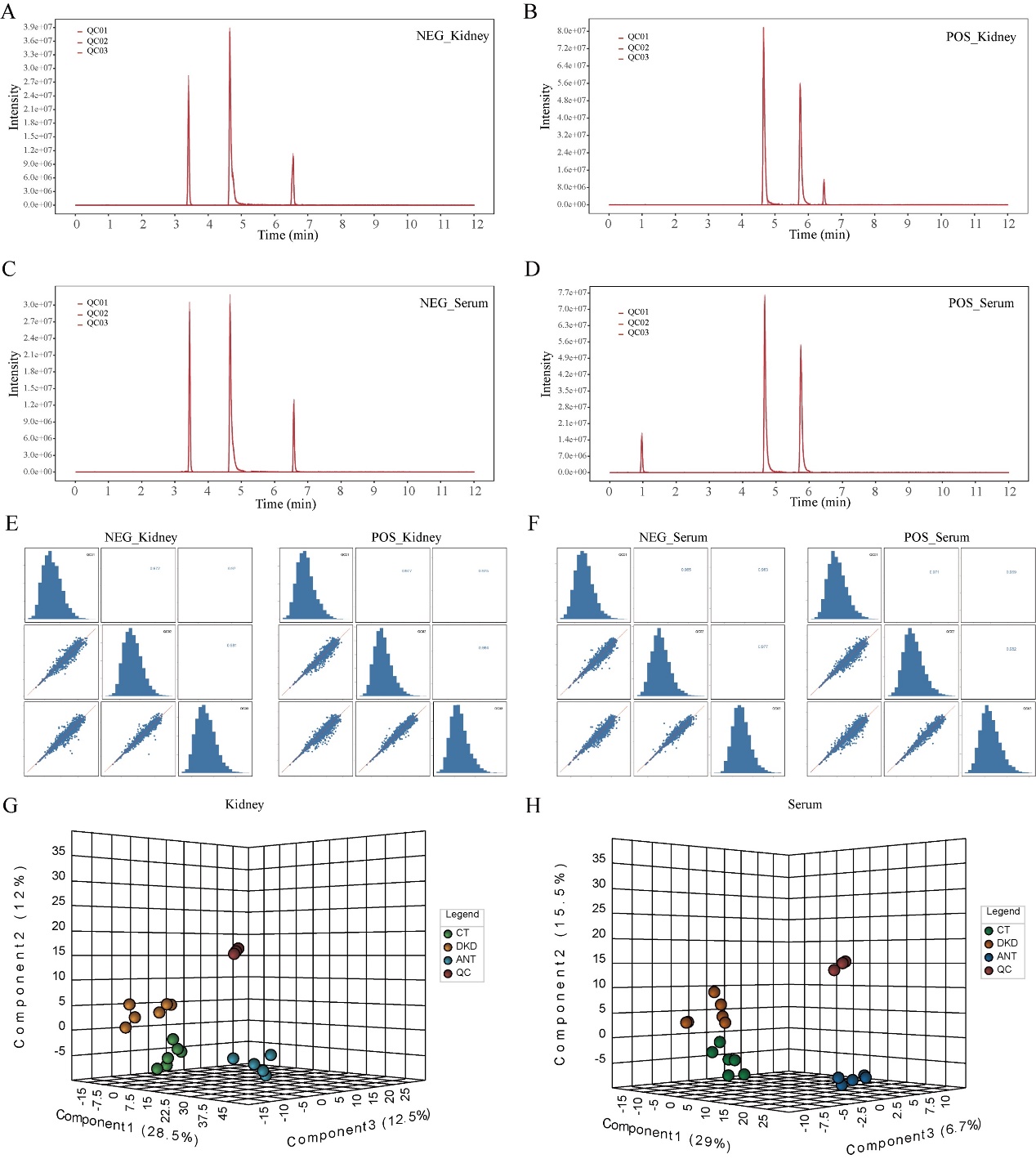
**

**Figure S2. The quality control (QC) of metabolomic profiling in the kidney and serum**

The extracted ion chromatograms (EIC) of the internal standard (IS) in kidney QC samples in negative (A) and positive (B) ion model. The EIC of the IS in serum QC samples in negative (C) and positive (D) ion model. Pearson correlation analysis for QC samples in the kidney (E) and serum (F). 3D-PLS-DA score plots exhibited a significant separation trend among the CT, DKD, ANT, and QC groups in the kidney (G) and serum (H).


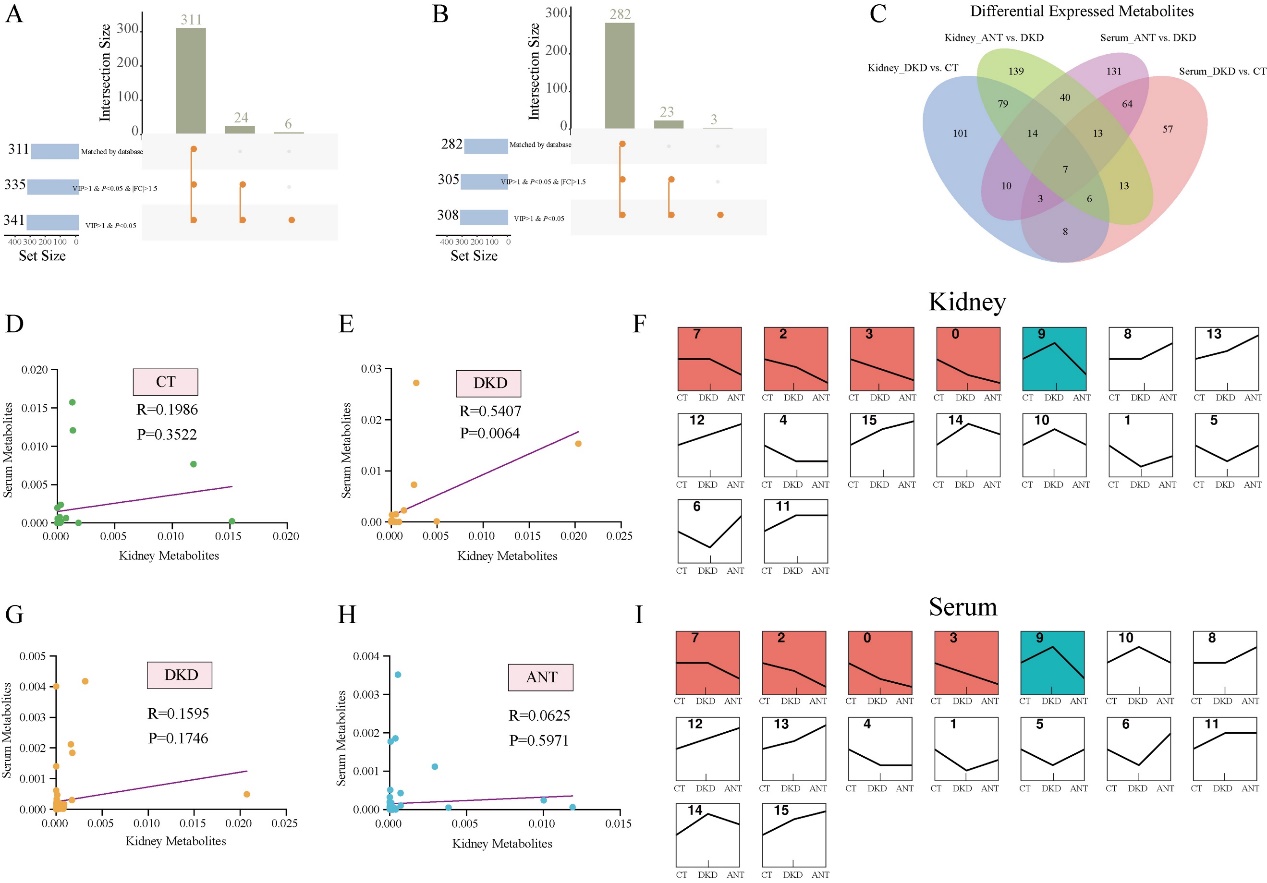


**Figure S3. Metabolic profile in the kidney and serum of the ANT group**

The upset plot for differentially expressed metabolites in the kidney (A) and serum (B) between the ANT and DKD groups. (C) Venn plot showing the intersection of significantly altered metabolites (FC > 1.5 or FC < 0.67, and *P* < 0.05) in the kidney and serum samples. Scatter plot showing the Pearson’s correlation of combined DEMs between serum and kidney metabolites in the CT (D) and DKD (E) groups. Scatter plot showing the Pearson’s correlation of combined DEMs between serum and kidney metabolites in the DKD (G) and ANT (H) groups. The kidney (F) and serum (I) metabolites profiles after STEM analysis. STEM analysis was applied to obtain the metabolite expression profiles across the CT, DKD, and ANT groups. Profile ID is shown at the top left corner of the profile. Lines in each profile represent the expression pattern of proteins across the three groups (permutation test, *P* < 0.05).

**
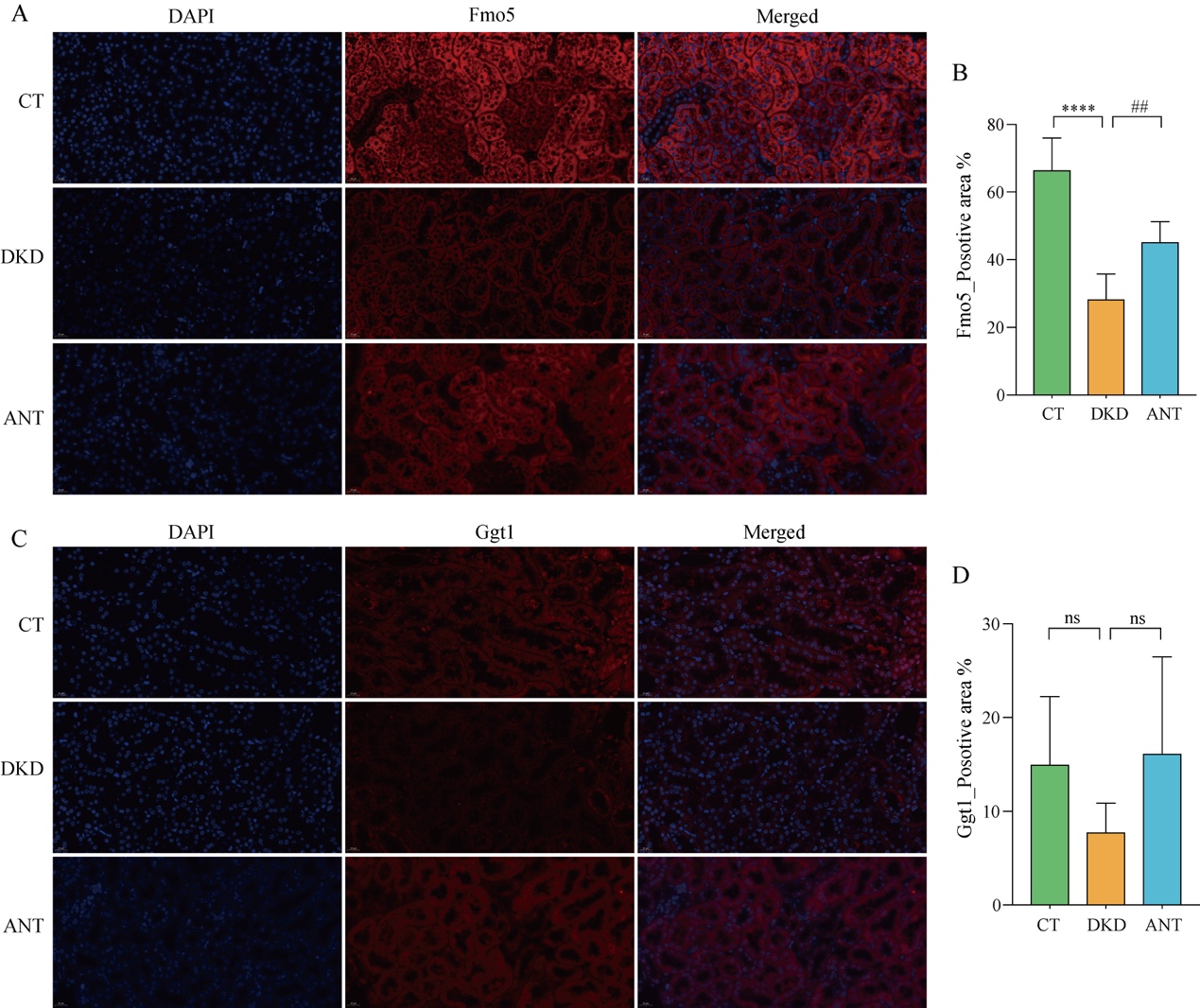
**

**Figure S4.** **Immunostaining assays of ANT-treated kidney samples**

Immunofluorescence revealed the expression of (A) Fmo5 and (C) Ggt1 levels in kidney tissues for CT, DKD, and ANT groups (blue fluorescence for DAPI; red immunofluorescence for Fmo5 or Ggt1; and scale bar as 20 µm). The immunostaining average positive area ratio of (B) Fmo5 and (D) Ggt1. The results of the statistical analysis are presented in bar charts (mean ± *SD*), by one-way ANOVA and multiple comparison using Holm-Sidak’s multiple comparisons test. **** *P* < 0.0001 compared with CT; ## *P* < 0.005 compared with DKD; ns, not significant.
